# From Hemorrhage to Diarrhea: The Comprehensive Clinical Journey of a Patient With Pseudomembranous Colitis

**DOI:** 10.7759/cureus.65176

**Published:** 2024-07-23

**Authors:** Fathima Nilofar, Nithesh Babu, Mahendra Kumar, Saranya Palanisamy, Gnanadeepan T

**Affiliations:** 1 General Medicine, Saveetha Medical College and Hospital, Chennai, IND

**Keywords:** fecal microbiota transplantation, relapse, multidisciplinary approach, metronidazole, vancomycin, diarrhoea, pseudomembranous colitis, clostridium difficile

## Abstract

Pseudomembranous colitis (PC) is an inflammation of the colon primarily caused by the bacterium Clostridium difficile (C. difficile), often following antibiotic use. This case report describes the intricate clinical course of a 48-year-old male farmer with a history of chronic alcoholism, tobacco use, and seizure disorder, who presented with acute onset of left-sided weakness. CT brain revealed an intra-axial hemorrhage in the right gangliocapsular region with significant edema and midline shift. The patient's condition necessitated mechanical ventilation due to a low Glasgow Coma Scale (GCS) score. Complications ensued with the onset of ventilator-associated pneumonia (VAP) on day six, attributed to multi-drug resistant Acinetobacter baumannii, which was managed with meropenem and polymyxin.

Following successful weaning from the ventilator, he experienced severe watery diarrhea, high-grade fever, and diffuse abdominal pain on day 13. Subsequent stool tests confirmed PC caused by C. difficile, characterized by diffuse colonic wall-thickening with a water target sign on contrast-enhanced CT (CECT) abdomen. Initial treatment with oral vancomycin and metronidazole was followed by symptomatic treatment. Two weeks later, the patient had a relapse of PC, presenting with multiple episodes of loose stools, which was managed with oral metronidazole alone. Colonoscopy and biopsy confirmed the relapse, showing inflamed colonic mucosa with pseudomembranes. This case highlights the importance of strict infection control, prudent antibiotic use, and close monitoring for these patients. It also suggests the potential role of fecal microbiota transplantation (FMT) for recurrent cases. The patient's recovery demonstrates the effectiveness of meticulous medical management and adherence to infection control protocols in achieving optimal outcomes.

## Introduction

Pseudomembranous colitis (PC), a severe inflammation of the inner lining of the large intestine, manifests as an antibiotic-associated colonic inflammatory complication. The disease most commonly results from a serious Clostridium difficile (C. difficile) infection, a nosocomial issue increasingly on the rise in the last two decades [1]. Antibiotics often alter the natural colonic mucosa, leading to C. difficile colonization, which causes endothelial damage, resulting in necrotic areas on the surface epithelium, followed by inflammation [2]. The less common non-C. difficile causes of PC should also be considered, as this condition is associated with several etiological factors. Examples include Behcet’s disease, collagenous colitis, inflammatory bowel disease, ischemic colitis, other infectious organisms (e.g., bacteria, parasites, viruses), and a handful of drugs and toxins.

The most common clinical manifestations of PC are bloody diarrhea, hypotension, tachycardia, fever, and abdominal tenderness [3]. The most severe cases of C. difficile infection manifest with significant complications, including systemic inflammatory response syndrome (SIRS), septicemia, intestinal paralysis, toxic megacolon, and colonic perforation [4]. Of note, 0.4-3% of cases may progress to fulminant colitis with toxic megacolon, which has a high mortality rate (38-80%) and requires surgical intervention [5]. We report a case of a 48-year-old male with a complex medical history, including chronic alcohol and tobacco use and a seizure disorder. His clinical course was further complicated by an acute hemorrhagic stroke, requiring intensive care management. Such severe neurological events often necessitate prolonged hospitalization and invasive procedures, which increase the risk of hospital-acquired infections, including ventilator-associated pneumonia (VAP) and PC.

The management of PC involves a multifaceted approach, including the cessation of the inciting antibiotics, initiation of targeted therapy with oral vancomycin or fidaxomicin, and supportive care. Recurrent infections pose a significant challenge, often requiring repeated or prolonged antibiotic therapy and sometimes fecal microbiota transplantation (FMT) for resistant cases. It is mandatory to isolate C. difficile-infected patients and to thoroughly clean environmental surfaces with a chlorine-based disinfectant to prevent the spread [6]. Through detailed documentation of this patient's clinical journey, we aim to contribute to the understanding of the complexities in managing PC in patients with multifaceted health issues, emphasizing the critical role of a multidisciplinary approach in improving patient outcomes.

## Case presentation

A 48-year-old male farmer from Tamil Nadu presented to the emergency room with a sudden onset of weakness in the left upper and lower limbs with a headache. The patient had a progressive difficulty in moving his left hand and leg associated with altered sensorium and difficulty in comprehending speech. He had a known history of seizure disorder for the past three years, although he was not on regular follow-up or treatment. His personal history revealed chronic alcohol and tobacco use for 20 years. There was no significant family history of stroke or other neurological disorders.

**Figure 1:**
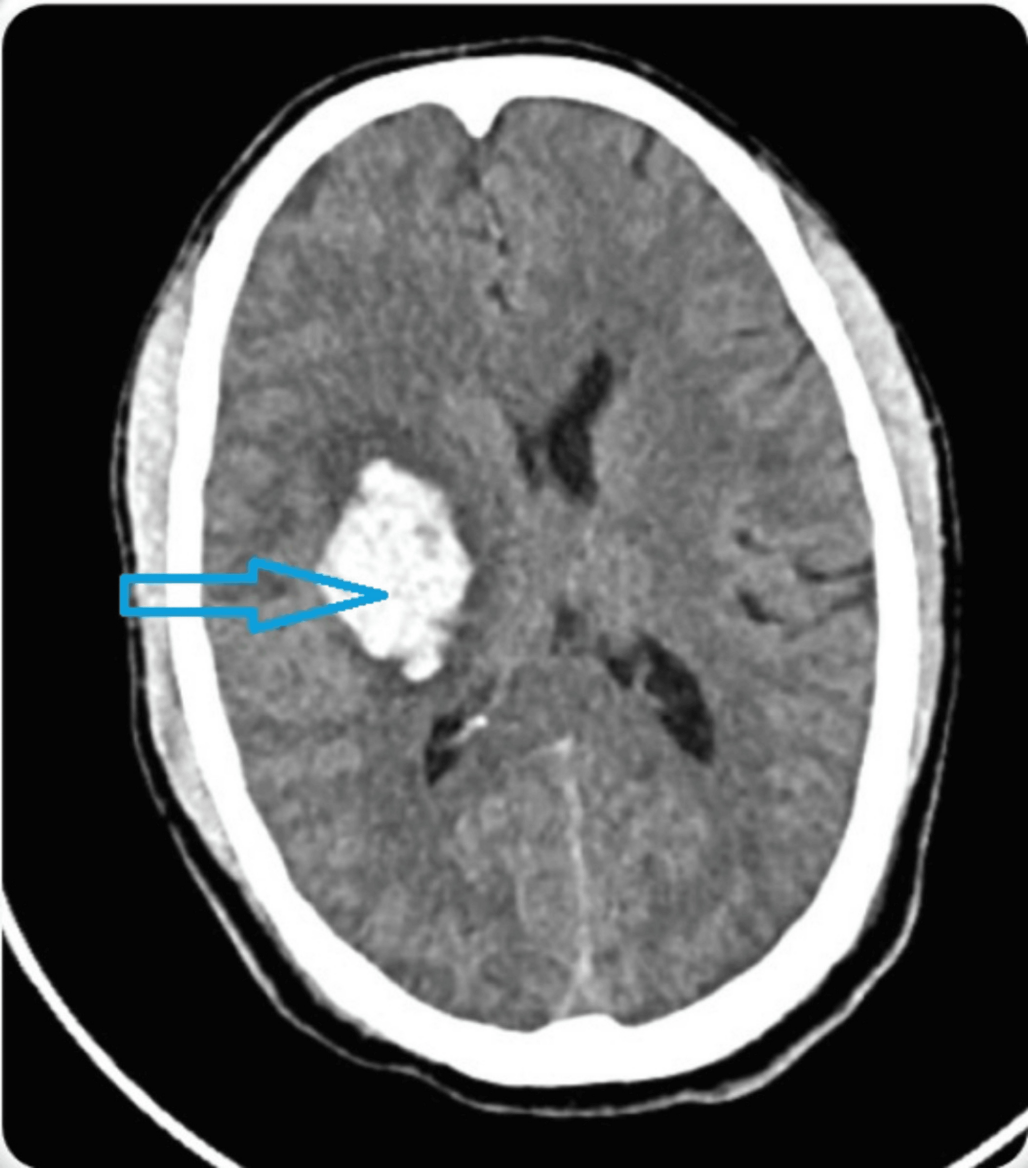
CT brain findings The blue arrow shows a well-defined supratentorial intra-axial hemorrhage measuring 3.9 x 2.9 x 3.5 cm in the right gangliocapsular region, extending to the corona radiata and centrum semiovale, with perifocal edema and midline shift CT: computed tomography

On examination, the patient’s vital signs indicated severe hypertension (BP: 200/110 mmHg), bradycardia (PR: 65/min), normal respiratory rate, normal oxygen saturation, and a Glasgow Coma Scale (GCS) score of 9 (E3V1M5) on arrival. The cranial nerve examination showed left-sided facial weakness, while motor examination indicated normal muscle bulk but decreased tone and complete paralysis (0/5 power) in the left upper and lower limbs. Reflexes were exaggerated on the left side with a positive Babinski sign, suggesting an upper motor neuron lesion. CT brain revealed a well-defined supratentorial intra-axial hemorrhage measuring 3.9 x 2.9 x 3.5 cm in the right gangliocapsular region, extending to the corona radiata and centrum semiovale, with perifocal edema and midline shift (Figure 1). He was admitted to the ICU and managed with anti-edema measures, antihypertensives, and anti-epileptics. Given the drop in GCS, he was intubated on day two. Serial CT scans were performed to monitor the progression of the hemorrhage and the patient was managed conservatively.

On day six, the patient, who was under mechanical ventilation, developed persistent fever spikes and was evaluated for VAP. Endotracheal culture and sensitivity (ET C/S) tests identified multi-drug resistant Acinetobacter baumannii, which was sensitive only to tigecycline and polymyxin. The patient was started on intravenous meropenem and polymyxin. Over the course of the treatment, the patient showed symptomatic improvement and was successfully weaned off the ventilator.

**Figure 2:**
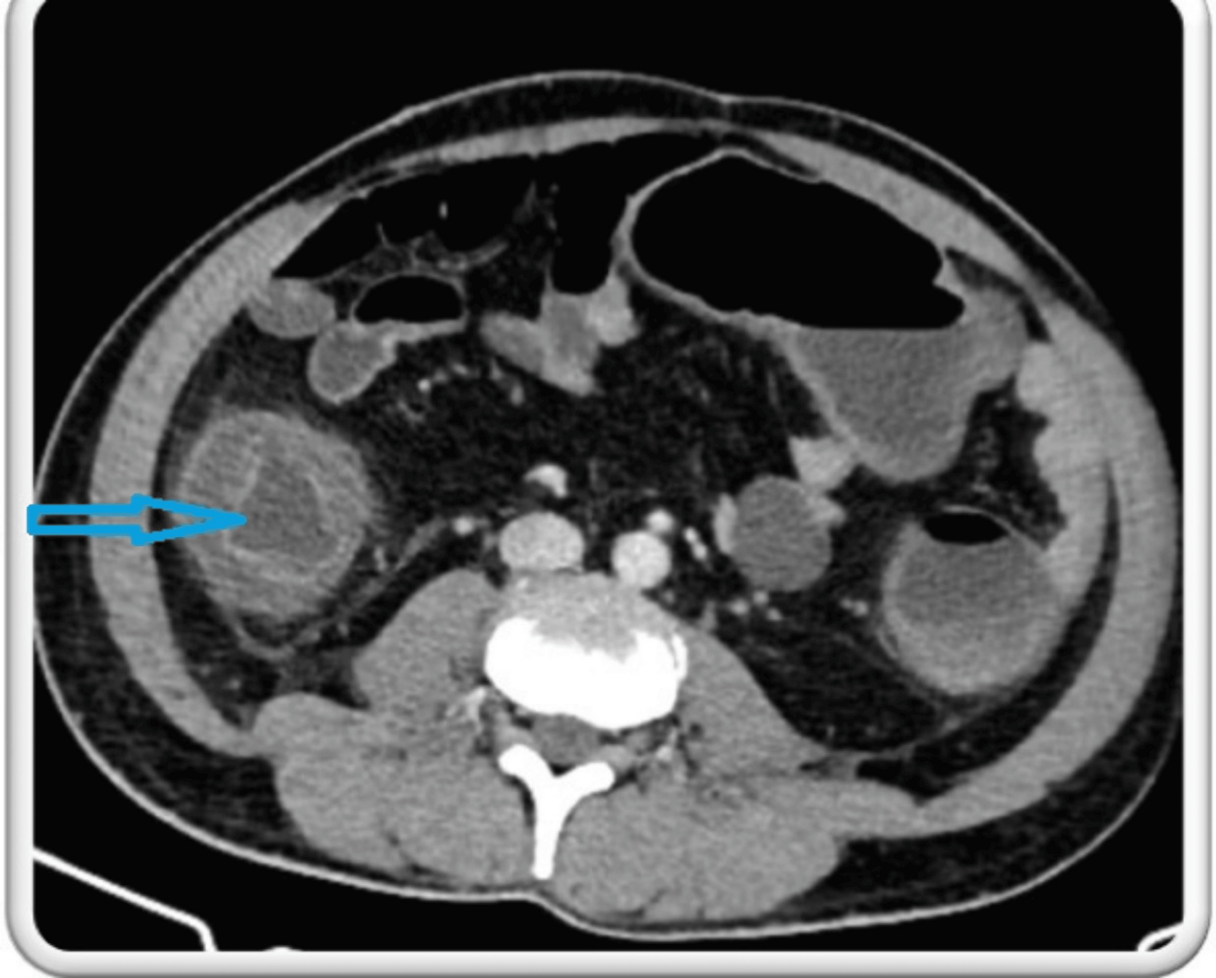
CECT of the abdomen The blue arrow shows diffuse symmetrical smooth circumferential mural wall-thickening with submucosal edema (water target sign) involving the entire colon, suggestive of pancolitis CECT: contrast-enhanced computed tomography

However, on day 13, he complained of multiple episodes of loose stools, which were watery and non-blood stained, accompanied by persistent high-grade fever and diffuse abdominal pain. His abdominal examination revealed diffuse tenderness, guarding, and rigidity, prompting the initiation of supportive management with nil per oral (NPO), Ryle's tube insertion, intravenous fluids, probiotics, and anti-secretory drugs. A contrast-enhanced CT (CECT) abdomen was performed, which showed diffuse symmetrical smooth circumferential mural wall-thickening with submucosal edema (water target sign) involving the entire colon, suggestive of pancolitis (Figure 2).

**Figure 3:**
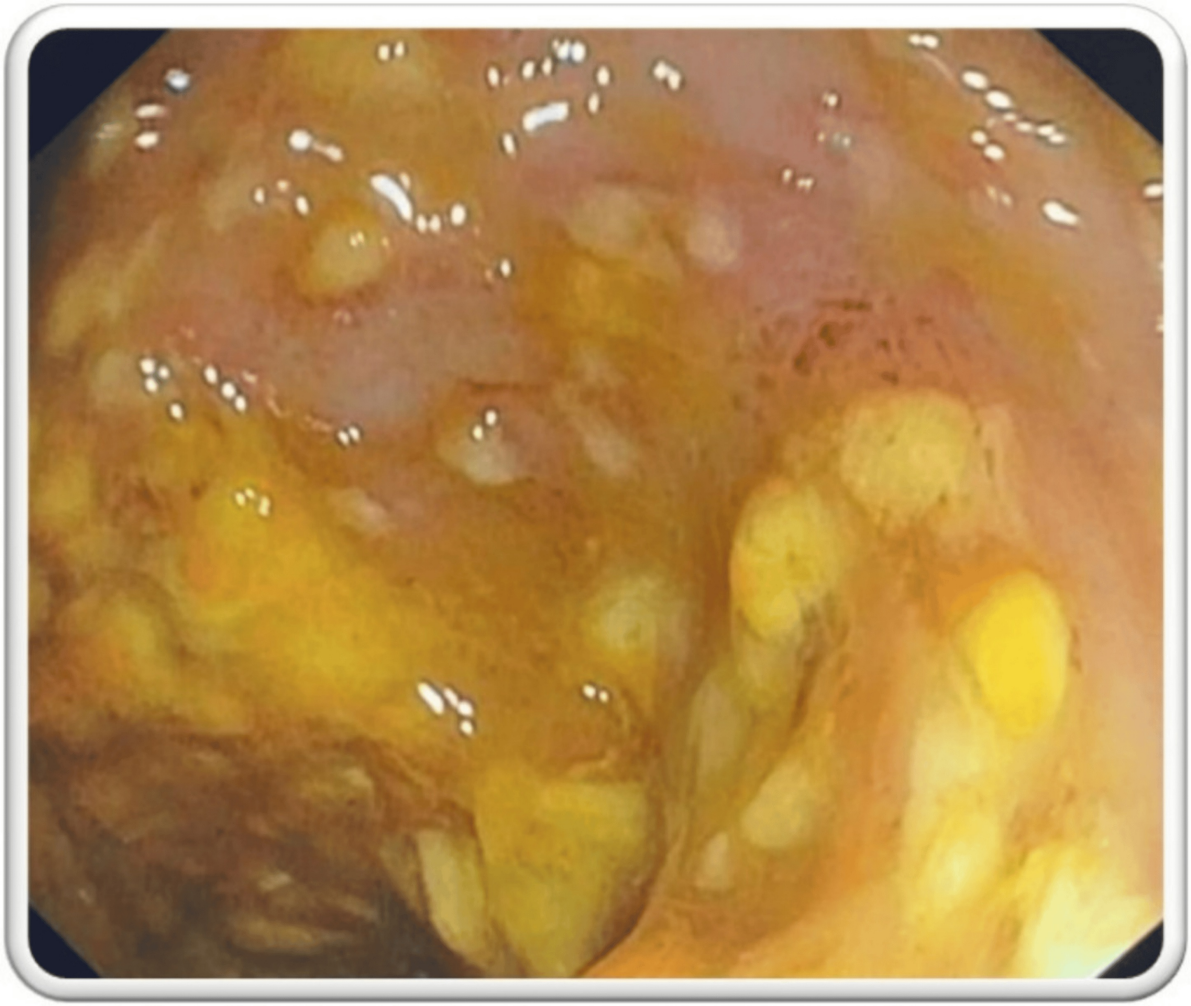
Colonoscopy demonstrating pseudomembranous colitis Diffuse pseudomembranes with erythematous changes, yellow-colored nodules, and hemorrhagic spots were seen studded in the entire colon

**Figure 4:**
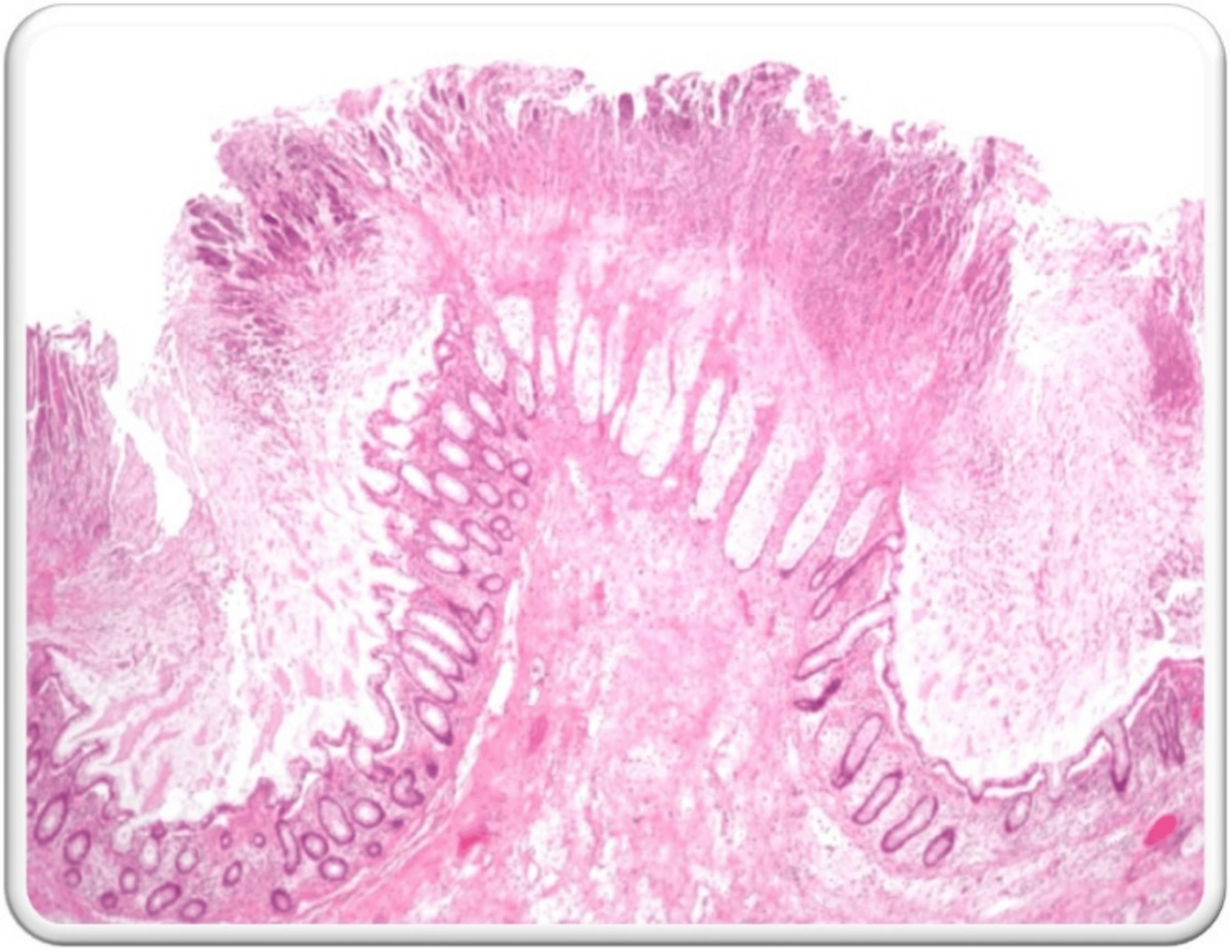
Histopathology report obtained from colonoscopy-guided biopsy Histopathology showed fragments of colonic mucosa with superficial mucosal congestion, mild lymphocytic and plasmacytic infiltration, and few neutrophils with no evidence of dysplasia. Pseudomembranous layers of exudate with erupting 'volcanic' crypts were seen, pointing more towards pseudomembranous colitis

Stool tests confirmed the presence of C. difficile toxins A and B, prompting a diagnosis of PC. The patient was treated with oral vancomycin and metronidazole for 14 days and kept in isolation with strict aseptic precautions in the ICU. He showed symptomatic improvement after five days of antibiotic therapy and was subsequently shifted to the ward. Despite initial recovery, he experienced a recurrence of loose stools after two weeks. This time, there was no associated abdominal pain or fever spikes. Supportive treatment with intravenous fluids, anti-secretory drugs, and probiotics was initiated. Due to the non-resolution of symptoms, a relapse was suspected, and a medical gastroenterologist's opinion was obtained. A colonoscopy and biopsy confirmed the diagnosis of relapsing PC. The colonoscopy revealed an inflamed colonic mucosa with pseudomembranes and raised yellowish nodules (Figure 3). Histopathology showed fragments of colonic mucosa with superficial mucosal congestion, mild lymphocytic and plasmacytic infiltration, and few neutrophils, with no evidence of dysplasia or malignancy (Figure 4).

The patient was restarted on metronidazole 500 mg orally three times daily, leading to improvement after three days, and no further episodes of loose stools were reported. Vancomycin was avoided the second time to prevent antibiotic resistance. The case highlights the critical importance of infection control measures, including antibiotic stewardship and comprehensive patient monitoring to prevent and manage severe complications like PC.

Preventive measures were reinforced, emphasizing the reduction of unnecessary antibiotic use, adherence to hand hygiene, and regular disinfection of high-touch surfaces in healthcare settings. The role of FMT was considered for recurrent cases unresponsive to standard antibiotic therapy. This case report underscores the complexities of managing patients with multifaceted health issues and the necessity of a multidisciplinary approach to improve patient outcomes. The patient’s recovery from a potentially fatal condition through meticulous medical management and adherence to infection control protocols demonstrates the significance of these strategies in clinical practice.

## Discussion

PC is characterized by inflammation of the colon, specifically the formation of pseudomembranes on the mucosal surface. The primary causative agent is the bacterium C. difficile, although other factors such as antibiotic use, underlying health conditions, and immunosuppression can also be contributory. PC typically arises in healthcare settings, where the disruption of normal gut flora allows C. difficile to proliferate and produce toxins (A and B), leading to colonic inflammation [7]. Antibiotics, such as clindamycin, penicillins, fluoroquinolone, and cephalosporins are typically associated with C. difficile infection, but almost any anti-bacterial agent can cause the disease.

PC is characterized by distinctive clinical features primarily associated with the inflammation of the colon. Common symptoms include severe and recurrent diarrhea, frequently watery and accompanied by abdominal cramps. The severity of PC can vary, ranging from mild discomfort to life-threatening conditions such as toxic megacolon. Notably, the hallmark clinical feature is the formation of pseudomembranes on the colonic mucosa, which can be observed during endoscopic examinations. These pseudomembranes consist of inflammatory debris, mucin, and white blood cells.

In severe cases, complications may arise, including perforation of the colon, sepsis, and SIRS [8,9]. The diagnosis of PC involves a combination of clinical evaluation and laboratory tests. Stool tests, specifically for the detection of C. difficile toxins (toxins A and B), are crucial in confirming the presence of this bacterium as the causative agent. Additionally, endoscopic examinations, such as sigmoidoscopy or colonoscopy, may reveal pseudomembranes on the colonic mucosa, providing a direct visualization of the characteristic inflammatory features. Biopsy samples from affected areas can further aid in confirming the diagnosis. A comprehensive approach involving clinical symptoms, laboratory results, and endoscopic findings is essential for accurate and timely identification of PC.

The treatment of PC primarily involves addressing the underlying C. difficile infection. Antibiotics such as vancomycin or metronidazole are commonly prescribed to target and eliminate bacterial overgrowth. In severe cases, FMT may be considered to restore a healthy microbial environment in the colon. Early recognition and appropriate management are crucial in achieving successful outcomes in the treatment of PC. Newer modalities like oral fidaxomicin can be considered for severe resistant cases. The management of nosocomial diarrhea, especially in cases like PC, requires a thorough and multidisciplinary approach. This case report underscores the importance of vigilant monitoring, timely interventions, and collaboration among various medical specialties for a successful patient outcome [10].

## Conclusions

This case report demonstrates the intricate challenges in managing a patient with significant comorbidities, including chronic alcoholism, tobacco use, and seizure disorder, who presented with an acute hemorrhagic stroke. The development of PC following broad-spectrum antibiotic therapy highlights the critical need for judicious antibiotic use and strict infection control measures. The patient's eventual recovery, following tailored antibiotic therapy and comprehensive supportive care, underscores the importance of a multidisciplinary approach in managing complex clinical scenarios. This case also emphasizes the potential role of FMT in recurrent C. difficile infections. Overall, this report sheds light on the necessity of vigilant monitoring, prompt intervention, and coordinated care in improving patient outcomes in similar multifaceted cases.
